# Feeding study for the mycotoxin zearalenone in yellow mealworm (*Tenebrio molitor*) larvae—investigation of biological impact and metabolic conversion

**DOI:** 10.1007/s12550-019-00346-y

**Published:** 2019-03-13

**Authors:** Kelly Niermans, Jan Woyzichovski, Nina Kröncke, Rainer Benning, Ronald Maul

**Affiliations:** 1 BfR - German Federal Institute for Risk Assessment, Max-Dohrn-Str. 8-10, 10589 Berlin, Germany; 2 University of Applied Sciences Bremerhaven, An der Karlstadt 8, 27568 Bremerhaven, Germany

**Keywords:** *Tenebrio molitor*, Yellow mealworm, Zearalenone, α-ZEL, β-ZEL, Phase II metabolites

## Abstract

Edible insects as additional food and/or feed source may represent one important component to solve the problem of food security for a growing human population. Especially for covering the rising demand for protein of animal origin, seven insect species currently allowed as feed constituents in the European Union are gaining more interest. However, before considering insects such as yellow mealworm larvae (*Tenebrio molitor*) as suitable for, e.g. human consumption, the possible presence and accumulation of contaminants must be elucidated. The present work investigates the effects of the mycotoxin zearalenone (ZEN) and its metabolites on insect larvae. Seven different diets were prepared: toxin-free control, spiked and artificially contaminated (both containing approx.500 μg/kg and approx. 2000 μg/kg of ZEN) as well as two naturally contaminated diets (600 μg/kg and 900 μg/kg ZEN). The diets were used in a multiple-week feeding trial using *T. molitor* larvae as model insects. The amount of ZEN and its metabolites in the feed, larvae and the residue were measured by HPLC-MS/MS. A significantly enhanced individual larval weight was found for the insects fed on the naturally contaminated diets compared to the other feeding groups after 8 weeks of exposure. No ZEN or ZEN metabolites were detected in the *T. molitor* larvae after harvest. However, ZEN, α- and β-stereoisomers of zearalenol were found in the residue samples indicating an intense metabolism of ZEN in the larvae. No further ZEN metabolites could be detected in any sample. Thus, ZEN is not retained to any significant amount in *T. molitor* larvae.

## Introduction

The continuous growth of the world population increases the global demand for food, land, water and energy (Godfray et al. 2010). Consequently, van Huis et al. (2013) stated that the food production must grow, while at the same time, the environmental impact of both agriculture and livestock must decrease (Foley et al. 2011; van Huis et al. 2013). A change of diet was one of the solutions proposed (Godfray et al. 2010). Edible insects were suggested as an additional food source due to their favourable nutritional properties including high protein, fat and mineral contents (Rumpold and Schluter 2013; van Huis 2013; van Huis et al. 2013). Additionally, insects reproduce quickly; have high feed conversion efficiency, a low environmental footprint and low water consumption; and there is a possibility to rear them on waste substrates (Bovera et al. 2015; van Huis et al. 2013). Therefore, the use of certain insect species can provide in the increasing demand for animal-derived proteins. These insect-derived proteins can additionally be used in feed processing and completely or partly substitute fishmeal and soy due to their current high prices (Bovera et al. 2015). Furthermore, the availability of fishmeal and soy in the future will be limited; therefore, alternatives must be found (Makkar et al. 2014).

Insects belong to the group of phylum Arthropoda, consist of a wide variety of animals and serve in different stages of the feed and food production chain. This diversity consequently displays the complexity and main challenge of implementing insects in the food and feed production chain (Van der Fels-Klerx et al. 2016; van Raamsdonk et al. 2017). Within the EU, insects supposed to be used as food are mostly under the Novel Food Regulation and therefore need authorization before allowance on the European market (European Parliament and the Council of the European Union 2015). Additionally for feed, the processed animal proteins of only seven farmed insect species may be used (European Commission 2017). The yellow mealworm (*Tenebrio molitor*), which is the larval stage of Darkling beetles, is one of the allowed species (European Commission 2017). Additionally, the European law lays down rules on the use of substrates, stating that substrates for feeding insects may only contain products of non-animal material or category 3 animal origin material (European Commission 2017; European Parliament and the Council of the European Union 2009a, b). In practice, insects are often nourished on cereal side products, which might contain high amounts of mycotoxins.

**Fig. 1:**
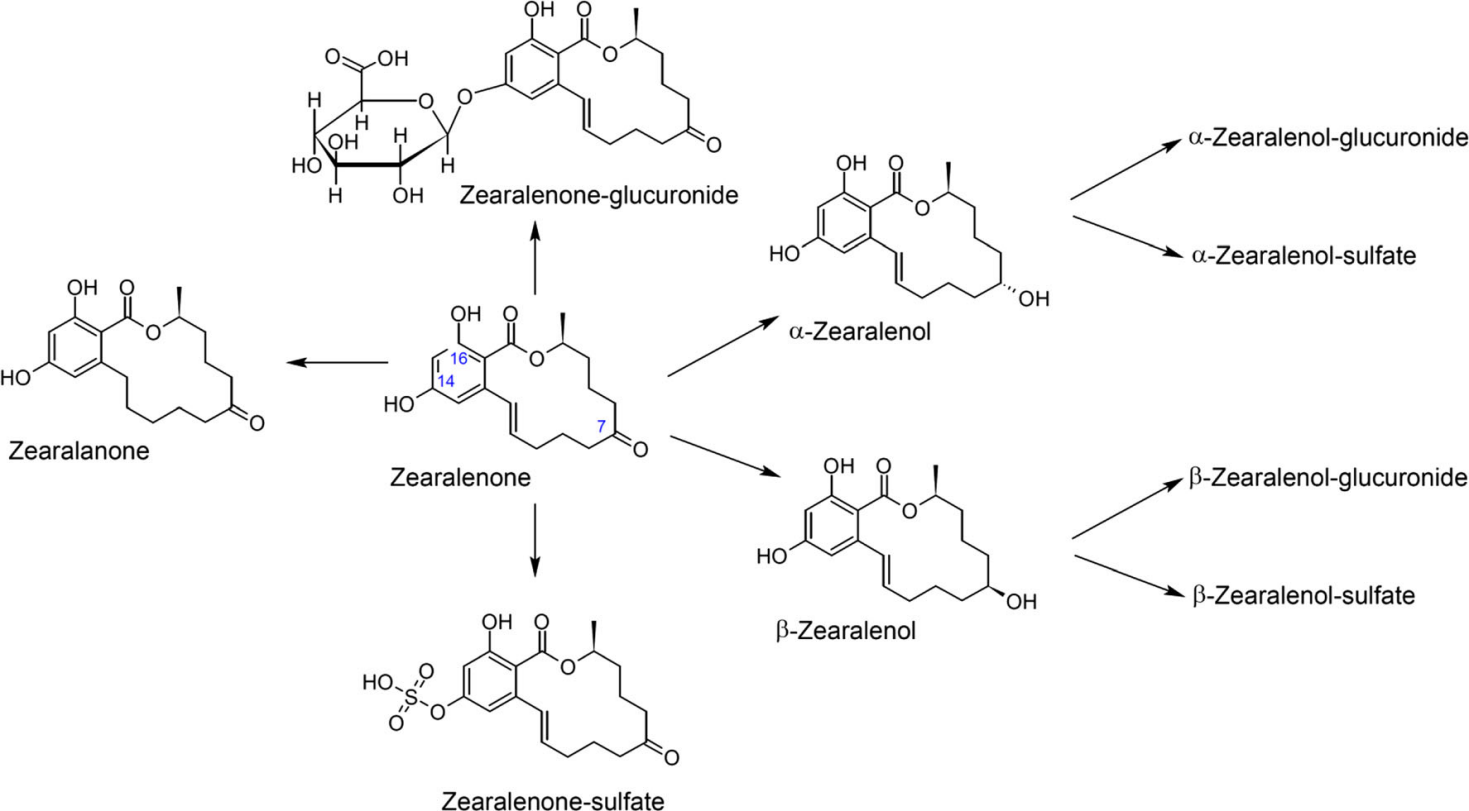
Metabolic pathway of zearalenone. Shown are only the C-14-linked glucuronides and sulfates. C-16 conjugates may also be formed and are known from in vitro studies (Metzler et al. 2010)

In this study, the focus is placed on zearalenone (ZEN) and its metabolites. ZEN is a macrocyclic β-resorcyclic acid lactone and is produced as a secondary metabolite by many *Fusarium* species (Hueza et al. 2014). A lot is already known on the effect and metabolism of ZEN in vertebrates. After ingestion, ZEN is metabolised by the intestinal tissue and hepatocytes. In the intestinal cells, the keto group on C-8 is reduced to α-, β-zearalenol (ZEL) and biotransformation into the metabolites α-, β-zearalanol (ZAL) and zearalanone (ZAN) is initiated (Fig. 1) (Hagler et al. 1979; Kinani et al. 2008; Metzler et al. 2010). ZEN and its metabolites are considered as endocrine disruptors due to their chemical structure which resembles the female hormone 17β-estradiol and makes oestrogen receptor binding possible (Kinani et al. 2008).

Additionally, ZEN and its metabolites may also be present in conjugated, e.g. sulfated form, either, insoluble and attached to macromolecules or as soluble conjugates (modified mycotoxins) (Berthiller et al. 2009; Binder et al. 2017; Keller et al. 2018; Rychlik et al. 2014; Warth et al. 2015). These modified forms can be obtained after metabolism or by transformation caused by chemical detoxification processes (Berthiller et al. 2013; Hussein and Brasel 2001; Warth et al. 2015). Excretion profiles obtained from previously conducted studies show that there is a significant difference in urinary excretion, glucuronidation or conjugation with sulfate of respectively deoxynivalenol (DON) and ZEN between animal species (EFSA 2017; Guerre 2015; Keller et al. 2018; Maul et al. 2012; Prelusky et al. 1986). Additionally, the metabolism of insects is known to be only distantly related to metabolic processes known from mammals or microorganisms (Binder et al. 2017; Ji et al. 2016; Keller et al. 2018; Malekinejad et al. 2006; Pfeiffer et al. 2007). Nevertheless, the presence of enzymes for phase I metabolism, e.g. cytochrome P450 monooxygenases or dehydrogenases as well as certain phase II enzymes such as sulfotransferases or glycosyltransferases, is described for insects as well (Ahn et al. 2012; Swevers et al. 1990; Vakiani et al. 1998). Furthermore, it is described that ZEN does not appear to have an obvious oestrogen-related target site in insects (Bhatnagar et al. 1991). However, high concentrations of ZEN have shown to contain weak antagonist properties towards ecdysone, a steroid hormone found in insects which regulates development and moulting (Dinan et al. 2001).

The oestrogenic activity of ZEN and its modified forms differ remarkably. Therefore, the European Food Safety Authority determined relative potency factors for ZEN and its modified forms. Compared to ZEN (set at 1.0), the relative potency factors of its metabolites are respectively 60 for α-ZEL, 4.0 for α-ZAL, 2.0 for β-ZAL, 1.5 for ZAN, 0.2 for β-ZEL. Additionally, the relative potency factors for glucuronides and sulfates are corresponding to their parent compound (EFSA 2016, 2017; Hagler et al. 1979; Metzler et al. 2010; Shier et al. 2001).

Contamination usually occurs in the field but can also occur during poor storage conditions of cereal-derived products. Maximum allowed levels for ZEN range from 50 μg/kg in processed grain products for human consumption to 350 μg/kg for unprocessed maize (EFSA 2011). ZEN was found in wheat and maize samples throughout Europe, in concentrations ranging from 15 μg/kg to as high as 5700 μg/kg after a year with a particularly wet summer (Hoogenboom et al. 2008; Schollenberger et al. 2006; Scudamore and Patel 2000). The maximum tolerable daily intake (TDI) of ZEN was previously established on 0.5 μg/kg body weight by the Joint FAO/WHO Expert Committee on Food Additives in 2000, and on 0.2 μg/kg body weight by the Scientific Committee on Food. More recently, the Panel on Contaminants in the Food Chain derived a TDI of 0.25 μg/kg body weight (EFSA 2011). Since 2016, the modified forms of ZEN were included in a group-TDI of 0.25 μg/kg body weight (EFSA 2016).

Previously performed studies showed that ZEN does not accumulate in the larval body of the lesser mealworm (*Alphitobius diaperinus*), which seems to be able to metabolise ZEN and excrete the mycotoxins (Camenzuli et al. 2018). Additionally, the nematode *Caenorhabditis elegans* appears to be able to metabolise zearalenone-14-sulfate (ZEN14Sulf) in α- and/or β-zearalenol-sulfate (ZELSulf) (Keller et al. 2018). However, comprehensive investigation on ZEL metabolism in *T. molitor* is lacking. Other studies addressing the effect of mycotoxins on insects include DON, aflatoxins and ochratoxin A (Bosch et al. 2017; Camenzuli et al. 2018; Van Broekhoven et al. 2017). Therefore, this study focusses on the impact of ZEN and some possible metabolites on *T. molitor*. Aims of the presented work were (i) to evaluate the overall recovery of ZEN as a further important *Fusarium* mycotoxin fed to *T. molitor* larvae in different diets, (ii) the evaluation of the effects on the larvae and (iii) the screening for potential metabolites.

## Materials and methods

### Materials and chemicals

Analytical standards were purchased from Romer Labs (Getzersdorf, Austria): ZEN and from Merck (Darmstadt, Germany): α-ZEL, β-ZEL, ZAN, β-ZAL, DON. ^13^C-labelled standards were purchased from Biozol (Eching, Germany): ZEN (certified purity 99.5%), DON (certified purity 99.5%), β-ZEL (certified purity 96%) and α-ZEL (certified purity 96%).

Methanol, acetonitrile (ACN), acetic acid (HAc), formic acid (FA), ammonium formate (NH_4_COOH) and magnesium sulfate (MgSO_4_) were purchased from Merck (Darmstadt, Germany). Double-deionised water was obtained through the use of a water-filtering machine (Milli-Pore, Merck).

### Samples

Mycotoxin-free whole-wheat grains were bought at a local supermarket (BIO Weizen) and milled to grain flour (ring sieve pore size: 0.5 mm) using an Ultra-Centrifugal mill ZM200 (Retsch, Haan, Germany). Artificially contaminated wheat flour was obtained as previously described by Borzekowski et al. (2018) for the inoculation of rice. In brief, moistened wheat grain flour was autoclaved and inoculated with *Fusarium graminearum* (strain F1 as described by Dubos et al. 2011) and incubated for 21 days. Subsequently, the flour was autoclaved to terminate the incubation, freeze dried and milled again for homogenisation. The resulting toxin-rich flour contained 541.4 mg/kg ZEN, 3.0 mg/kg α-ZEL, 11.4 mg/kg β-ZEL, 6.4 mg/kg ZAN and 3.4 mg/kg β-ZAL respectively (Borzekowski et al. 2018). Additionally, a wheat lot naturally contaminated with ZEN was provided by the European Reference Laboratory for mycotoxins (Geel, Belgium). The wheat was milled as described above and contained 0.84 mg/kg ZEN.

### Diet preparation

The control, artificially and naturally contaminated wheat flour samples used for the insect feeding were either spiked or mixed in different ratios to obtain seven diets and contained different amounts of ZEN as is shown in Table 1. Spiked feed S1 and S2 was obtained by spiking blank wheat flour with toxin solution at two different concentration levels. A1 and A2 were obtained from blending blank wheat flour with two different amounts of the high concentrated artificially contaminated wheat flour, and N1 was obtained from blending the naturally contaminated wheat flour with the blank wheat flour. The samples were homogenised in by shaking overnight in a turbula mixer (WAB GmbH, Nidderau-Heldenbergen, Germany) and considered homogenous as two random samples showed less than 20% deviation for the content. Additionally, the amount of starch present in the wheat samples was measured with the ENZYTEC™ starch kit (E1268) obtained from R-Biopharm (Darmstadt, Germany). Protein content was determined by use of the Kjeldahl method.

**Table 1:** Feed composition and amounts of deoxynivalenol (DON), zearalenone (ZEN), α-zearalenol (α-ZEL) and β-zearalenol (β-ZEL) present in the different feed types prepared

Wheat composition		Code	DON (μg/kg)	ZEN (μg/kg)	α-ZEL (μg/kg)	β-ZEL (μg/kg)	Protein (g/100 g)	Starch (g/100 g)
Blank control		C	572.0	<LOQ	nd	nd	9.9	53.6
Blank spiked	Low	S1	568.4	588.5	nd	nd	9.9a	53.6^a^
Blank spiked	High	S2	576.5	2254	nd	<LOQ	9.9^a^	53.6^a^
Blank + artificially contaminated	Low	A1	939.0	427.0	nd	11.3	9.9^b^	50.7^b^
Blank + artificially contaminated	High	A2	2101	2283	nd	76.2	9.9	50.7
Blank + naturally contaminated	Low	N1	2854	602.3	nd	<LOQ	11.5^b^	47.0^b^
Naturally contaminated	High	N2	4588	919.3	nd	<LOQ	12.3	44.5

*nd* not detected, therefore either not present or not present in detectable levels (LOQs are ZEN: 10.9 μg/kg; DON: 67.1 μg/kg, α-ZEL: 11.2 μg/kg; β-ZEL LOQ was comparable to α-ZEL and not determined separately)

^a^Not measured, however composed of the blank material by spiking, therefore assumed to be the same

^b^Not measured directly, however calculated according to the blending protocol of two samples with measured content

### Preparation stock and working solutions

Both a standard mix (ZEN, α-ZEL, β-ZEL, ZAN, β-ZAL, DON) and an isotopically labelled standard (IS) mix (ZEN, α-ZEL, β-ZEL) were prepared in ACN 1% HAc. The standard mix diluted by ACN 1% HAc served as calibration mix as well as spiking solution for validation. Calibration solutions were prepared by adding 180 μL H_2_O, 160 μL ACN 1% HAc, 20 μL of one out of eight calibration stock solutions and 40 μL of the IS solution.

### Larval selection, exposure and harvest

*T. molitor* larvae (family of Tenebrionidae), of Hochschule Bremerhaven’s own breeding, were initially kept on wheat bran as substrate and selected at an age of 42 days and on medium size (approx. 1 cm). Feeding groups, each containing 100 individuals, with an average weight of 7.80 ± 0.9 mg per individual were assembled in clear 400 mL glass beakers and incubated at 25 °C with a humidity of 75% with no day/night rhythm for light, temperature and humidity.

Exposure time was either 4 weeks (short-term, 70 days old larvae) or 8 weeks (long-term, 98 days old larvae). Each feeding group was fed with portions of 5–6 g of feed as soon as the feed in the beaker had been consumed. The exact amount of feed was recorded separately for every insect group. Biological triplicates were obtained for the seven diets prepared. Biological triplicates of the short-term exposed samples consisted of 200 individuals in order to obtain sufficient sample amount for further mycotoxin analysis (i.e. three times two groups were pooled) whereas biological triplicates of the long-term exposure included 100 individuals. Thus, for short-term as well as for long-term exposure for each of the seven diets, three replicate larvae and residue samples were obtained for analysis. After harvest and fasting for 24 h, the larvae were stored at − 18 °C before freeze drying in a Beta 1–8 LDplus freeze dryer (CHRIST, Osterode am Harz, Germany). The residue (combination of faeces and residual feed present in the beakers after the experiments) was kept separately and analysed for its mycotoxin content.

### Sample extraction

The extraction methods used are based on a pre-norm non-quantitative high-performance liquid chromatography (HPLC) tandem mass spectrometry (MS/MS) screening method developed by CEN/TC 275/WG5 in project number 05701704.

### Wheat extraction

The wheat was grinded in a Retsch Ultra-Centrifugal mill ZM200 (Haan, Germany) at a particle size of 0.5 mm. Homogenised dry wheat meal (2.5 g) was weighed in duplicates into a 50-ml Falcon tube and extracted using 5 ml H_2_O and 5 ml ACN 0.1% FA. Following, the samples were shaken for 30 min, centrifuged (30 min; 5833×*g*; 10 °C) and 1 ml of supernatant was taken out to which 100 μl IS and 100 μl H_2_O was added. Phase separation of organic and water phase was achieved by addition of 250 mg anhydrous MgSO_4_, shaking for 30 s (VXR B, IKA) and centrifugation at 17,530×*g* for 10 min at 10 °C (Microfuge R, Beckmann). Supernatant (300 μl) was transferred into a HPLC crimp vial, mixed with 300 μl H_2_O and either directly measured or stored at 3 °C for up to 6 days.

### Larvae/residue sample extraction

Larval samples were grinded in a Planetary Micro Mill, Pulverisette 7 premium line (FRITSCH GmbH, Idar-Oberstein, Germany). A variation on the method for multi mycotoxin extraction in wheat was used for larval and residue extractions. Two hundred milligrams of either grinded larvae or residue was weighed in duplicates into a 7-ml glass vial and extracted using 1.5 ml H_2_O and 1.5 ml ACN 0.1% FA. Following, the samples were mixed for 10 min in an ultrasonic bath, shaken for 30 min, centrifuged (30 min; 5833×*g*; 10 °C) and 1 ml of supernatant was taken out to which 100 μl IS and 100 μl H_2_O was added. Phase separation of organic and water phase was achieved by addition of 250 mg anhydrous MgSO_4_, shaking for 30 s (VXR B, IKA) and centrifugation at 17,530×*g* for 10 min at 10 °C (Microfuge R, Beckmann). Three hundred microliters of supernatant was transferred into a HPLC crimp vial, mixed with 300 μl H_2_O and either directly measured or stored at 3 °C for up to 6 days. Due to varying dilutions used, the LOQs for this method were calculated to be DON: 251 μg/kg, ZEN: 40.9 μg/kg; α-ZEL is 42.0 μg/kg. Additionally, the LOD for DON is 76.1 μg/kg, for ZEN is 12.4 μg/kg and for α-ZEL is 12.8 μg/kg.

### Recovery

Spiking experiments at a level of 2 mg/kg DON and 200 μg/kg ZEN were performed to determine the recovery within larval and residue samples. Recovery rates found in the larval samples were determined as follows: DON 92 ± 6% and ZEN 92 ± 4%. Additionally, recovery rates of 98 ± 6% for DON and 94 ± 4% for ZEN were found in the residue samples.

### HPLC-MS/MS instrumentation and parameters

Analyses of wheat, larvae and residue samples was performed on an Agilent 1290 series HPLC system (Waldbronn, Germany) coupled to a Q-Trap 6500+ system (AB Sciex, Foster City, CA, USA) equipped with an IonDrive™ Turbo V electrospray ionisation (ESI) source. Analytes were separated on a polar guard-coated C18 column, 100 × 2.1 mm, 5 μm, (Protecol, SGE). Eluent A consisted of H_2_O, 0.1% FA and 300 mg/l NH_4_COOH, eluent B was composed of methanol, 0.1% FA and 300 mg/l NH_4_COOH. After an initial period of 0.8 min at 15% B, the proportion of B was linearly increased to 60% at 4.0 min, 65% at 6.0 min, 80% at 8.5 min. At 11.0 min, the proportion of B reached 95% which was held until 12.0 min. At 12.5 min, the proportion of B was lowered to 15%. At a total time of 15.0 min, the method ended. The flow rate was set to 600 μl/min, the column temperature was 35 °C, and the injection volume was 4 μl. Mass spectrometric detection was performed in positive and negative ESI mode, and selected reaction monitoring was applied as scan type. For most substances, either reference standards were available (ZEN, α-ZEL, β-ZEL, ZAN, β-ZAL, DON) or matrix samples known to contain the respective analytes ZEN14Sulf, ZEN-14-*O*-glucoside, ZEN-16-*O*-glucoside and α-ZELSulf were analysed for verification of the MS parameters. Additionally, selected reaction monitoring transitions were incorporated for the analysis of hydrolysed zearalenone, decarboxylated hydrolysed zearalenone as well as ZEN- and ZEL-glucuronides based on literature data. The ESI-MS/MS parameters used are shown in Table 2.

**Table 2:** Overview of selected reaction monitoring parameters

Analyte	[M-H]^−^ (*m/z*)	DP [V]	Product ions Q/q (*m/z*)	CE [eV]	Reference(s)
Zearalenone (ZEN)	317.1	− 110	175.0/131.1	− 34/− 42	
ZEN-14-*O*-glucuronide	493.0	− 100	131.0/175.0	− 68/− 42	Warth et al. (2012)
ZEN-14-sulphate (ZEN14Sulf)	397.1	− 115	317.0/131.0	− 34/− 58	Binder et al. (2017)
ZEN-14-*O*-glucoside	479.1	− 125	317.1/175.0	− 22/− 54	Binder et al. (2017)
ZEN-16-*O*-glucoside	479.2	− 140	149.0/160.8	− 54/− 54	Binder et al. (2017)
Hydrolysed ZEN	335.0	− 30	149.0/161.0	− 30/− 30	Vekiru et al. (2016)
Decarboxylated hydrolysed ZEN	291.1	− 30	149.0/161.0	− 30/− 30	Hahn et al. (2015)
α-zearalenol (α-ZEL)	319.1	− 115	160.0/174.0	− 44/− 50	
α-ZEL-14-*O*-glucuronide	495.1	− 110	319.0/112.8	− 38/− 28	Binder et al. (2017)
α-ZEL-sulphate (ZELSulf)	399.2	− 50	319.2/275.2	−25/− 40	Brodehl et al. (2014)
β-zearalenol (β-ZEL)	319.2	− 115	174.0/160.0	− 50/− 44	
β-ZEL-14-*O*-glucuronide	495.1	− 110	319.0/112.8	− 38/− 28	Binder et al. (2017)
α-/β-zearalanol (ZAL)	321.2	− 75	277.1/303.2	− 30/− 25	
Zearalanone (ZAN)	319.2	− 75	275.1/205.0	− 35/− 40	
Deoxynivalenol (DON)	355.1	− 70	265.2/59.2	− 22/− 40	

### Data analyses

Wheat, larvae and residue samples were analysed in duplicates. Analyst Software version 1.6.3 and Multiquant version 3.0.2 (Sciex, Foster City, CA, USA) were used for analyses and quantification of the data obtained. Normalisation of the data was performed for the amount of mycotoxins found in the feed/residue and weight gain of the larvae in relation to the protein content of the feed. Data for larval weight gain was tested for significance with a one-way ANOVA test followed up by a Tukey-Kramer post hoc analysis and was performed by using Excel. The calculation of the total recovery of ZEN is based on measured contents, the total amount of residue (containing faeces and not consumed feed) collected and final weight of the larvae. Recovery rates were calculated for the amount of ZEN present in the feed in relation to the amount of ZEN, α- and β-ZEL present in the residue (see Table 3 for detailed short-term exposure data and Table 4 for long-term exposure data).

**Table 3:** Mean, standard deviation and recovery of the amount of ZEN, α- and β-ZEL in μg per absolute amount of feed, larvae and residue detected after 4 weeks of exposure and 24 h of fasting. The toxin amount in feed was calculated individually based on the measured content (Table 1) and the individually fed amount

	Feed (μg per amount feed)			Worm			Residue (μg per amount residue)				
Diet code	ZEN	α-ZEL	β-ZEL	ZEN	α-ZEL	β-ZEL	ZEN	α-ZEL	β-ZEL	Recovery (%) ZEN	Recovery (%) ZEN. α-ZEL and β-ZEL
C	0.0 ± 0.0	nd	nd	nd	nd	nd	nd	nd	nd	na	na
S1	20.0 ± 0.9	nd	nd	nd	nd	nd	7.8 ± 0.3	0.2 ± 0.1	1.0 ± 0.2	39	45
S2	76.6 ± 2.8	nd	0.1 ± 0.1	nd	nd	nd	32.7 ± 1.3	3.4 ± 0.3	6.3 ± 0.4	43	55
A1	14.5 ± 2.5	nd	0.4 ± 0.1	nd	nd	nd	3.6 ± 0.2	0.5 ± 0.1	1.8 ± 0.1	25	41
A2	77.6 ± 11.5	nd	1.6 ± 0.5	nd	nd	nd	15.6 ± 0.3	3.6 ± 0.5	8.0 ± 0.4	20	35
N1	20.5 ± 0.6	nd	0.2 ± 0.1	nd	nd	nd	3.4 ± 0.6	0.7 ± 0.2	1.7 ± 0.1	17	28
N2	31.3 ± 3.5	nd	0.4 ± 0.1	nd	nd	nd	4.8 ± 0.3	1.6 ± 0.3	2.9 ± 0.2	15	30

*nd* not detected, therefore either not present or not present in detectable levels (LOQs for feed were ZEN: 10.9 μg/kg and α-ZEL: 11.2 μg/kg. LOQs for worm and residue samples were ZEN: 40.9 μg/kg and α-ZEL is 42.0 μg/kg. In both cases, the LOQ of β-ZEL was comparable to α-ZEL and was not determined separately); *na* not applicable

**Table 4:** Mean, standard deviation and recovery of ZEN, α- and β-ZEL in μg per absolute amount of feed, larvae and residue detected after 8 weeks of exposure and 24 h of fasting. The toxin amount in feed was calculated individually based on the measured content (Table 1) and the individually fed amount

	Feed (μg per amount feed)			Worm			Residue (μg per amount residue)				
Diet code	ZEN	α-ZEL	β-ZEL	ZEN	α-ZEL	β-ZEL	ZEN	α-ZEL	β-ZEL	Recovery (%) ZEN	Recovery (%) ZEN. α-ZEL and β-ZEL
C	nd	nd	nd	nd	nd	nd	nd	nd	nd	na	na
S1	20.6 ± 0.9	nd	nd	nd	nd	nd	11.6 ± 0.3	0.3 ± 0.2	2.0 ± 0.4	56	67
S2	78.9 ± 2.9	nd	0.1 ± 0.1	nd	nd	nd	49.6 ± 1.5	3.8 ± 0.3	9.0 ± 1.1	63	79
A1	14.9 ± 2.5	nd	0.4 ± 0.1	nd	nd	nd	6.0 ± 0.4	1.2 ± 0.1	4.2 ± 0.4	40	77
A2	79.9 ± 11.9	nd	1.7 ± 0.5	nd	nd	nd	26.2 ± 2.2	6.8 ± 0.7	17.3 ± 2.1	33	63
N1	21.1 ± 0.6	nd	0.2 ± 0.1	nd	nd	nd	4.8 ± 0.5	1.8 ± 0.4	3.6 ± 0.5	23	49
N2	32.2 ± 6.6	nd	0.4 ± 0.1	nd	nd	nd	6.0 ± 0.4	3.8 ± 0.4	6.2 ± 0.7	19	50

*nd* not detected, therefore either not present or not present in detectable levels (LOQs for feed were ZEN: 10.9 μg/kg and α-ZEL: 11.2 μg/kg. LOQs for worm and residue samples were: ZEN: 40.9 μg/kg and α-ZEL is 42.0 μg/kg. In both cases, the LOQ of β-ZEL was comparable to α-ZEL and was not determined separately) ; *na* not applicable

## Results

### Larval weight gain and survival

In order to obtain a first impression whether the presence of ZEN has a negative effect on the health status of the insects, the weight gain and survival rates for the different feeding groups have been recorded. For all treatment groups, no significantly altered mortality rates could be observed. Neither in the treatment nor in the control groups dead insects were found.

**Fig. 2:**
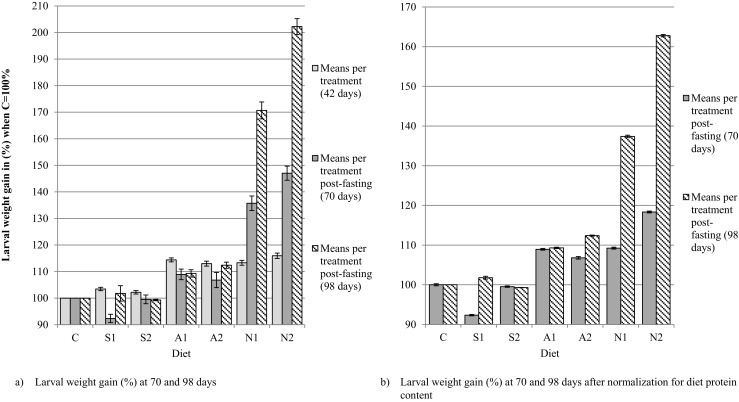
Means of larval weight gain in % of the control (C = 100%) during the experimental period

However, the average weight increase of the larvae did differ between diets provided. Additionally, after correction for the protein content of the diets administered, larvae fed with diet N1 and N2 gained significantly more weight than larvae fed with the other diets after 8 weeks of exposure (*p* < 0.05). The 8-week exposure of initially 6-week old larvae led to a difference in weight gain of not more than 12% compared to the control for all spiked and artificially contaminated feed groups. For the larvae fed with naturally contaminated flour, the mean weight gain was significantly (*p* < 0.05) increased with an increase in weight of 37% for N1 and 62% for N2 compared to the control after 8 weeks of exposure. Figure 2a summarises the absolute differences in weight gain for all feeding groups. In order to take into account the different protein amounts in the diets as an important factor for larval growth and also for weight gain in Fig. 2b, the relative increase in weight is corrected for the higher amount of protein available in the diet (i.e. 16% for N1 and 25% for N2). Still, clear difference between the N1 and N2 and the other groups after normalisation for the protein content is visible.

### ZEN and its reductive metabolites

ZEN and its metabolites were not present in detectable levels in the larvae after both short- and long-term exposure (Tables 3 and 4). On the other hand, detectable levels of ZEN as well as α- and β-ZEL were present in all residue samples except for the control samples. The detected amount of unmetabolised ZEN in the residues accounted for 15% up to 63% of the total ZEN amount that was fed to the respective insect groups. However, the reductive ZEN metabolites α- and β-ZEL together account for up to another 30% of the overall ZEN intake that is recovered in the residual samples. In all cases, the quotient of α- and β-ZEL was varying from 0.2 and 0.6 indicating an almost constant ratio of metabolites resulting from the metabolic conversion in the *T. molitor* model.

Based on the mass balance data of ZEN, it becomes clear that spiked samples S1 and S2 show a higher recovery rate than artificially and naturally contaminated samples. In the 4-week feeding trial, the overall relative amount of ZEN that is recovered in the residues either as unchanged ZEN or in its reduced forms is lower than in the 8-week experiment.

**Fig. 3:**
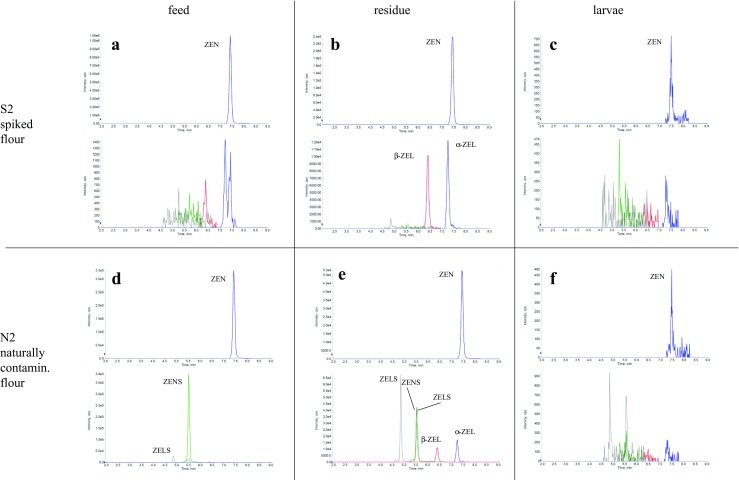
Chromatograms of ZEN and ZEL presence in the feed and residue of diets S2 and N2 and the presence of ZEN14Sulf and α- and/or β-ZELSulf in the same samples. Chromatograms are shown for the 8-week exposure feeding S2 and N2 exemplarily. After 4 weeks of feeding and for the diets S1 and N1 comparable but less pronounced signals could be observed

Notable is the fact that α- and β-ZEL were detected in all residue samples, while they did not occur in all feed samples as it is shown in Fig. 3a, b exemplarily for the 8-week feeding trial using S2. The detected levels for α- and β-ZEL were higher in the 8-week exposed samples compared to the 4-week exposed samples.

### Phase II metabolites

ZEN14Sulf is detectable in the naturally contaminated feed N1 and N2 as well as in the artificially contaminated feed (A1 and A2), while this compound is not detectable in the spiked or control feed (C, S1 and S2). Additionally, at least one ZELSulf signal was detectable in N1, N2 and A2 feed. No further conjugated ZEN metabolites are present in any feed. In none of the *T. molitor* samples, any glucuronide or sulfate signals are detectable. However, regardless the exposure time of 4 or 8 weeks, in the residues of groups N1, N2 and A2 clear peaks for ZEN14Sulf as well as two different peaks for ZELSulf were detectable. By contrast, in no residue sample collected for the spiked feed groups (S1 or S2) or the control, any phase II metabolite peak of ZEN was detectable, indicating that within the *T. molitor* organism, no sulfation occurs (Fig. 3b). Nevertheless, the relative intensity of the ZEN14Sulf and ZELSulf signals shifted from the more intense ZEN14Sulf in feed towards the reduced metabolite ZELSulf in the residue (Fig. 3a, e). Neither for ZEN or ZEL glucosides nor the respective glucuronides, any signals could be observed in any of the HPLC-MS/MS analyses.

### Other mycotoxins

As besides ZEN and ZEN14Sulf also the often co-occurring DON was present in the naturally contaminated flour and in the control flour, measurements have been carried out in all feed, *T. molitor* and residue samples for this toxin as well. Additionally, mass transitions specific for hydrolysed zearalenone and decarboxylated hydrolysed zearalenone as known degradation products of ZEN as well as ZAN and β-ZAL transitions were included in the HPLC-MS/MS method. For none of the aforementioned ZEN metabolites, any signals could be detected in the *T. molitor* samples. Also, no detectable amounts of DON were present in the larvae (Table 5). While also in the residual samples, no detectable amounts of any of the ZEN metabolites were present, DON could be detected and quantified in all residue analyses. Concerning the mass balance, the recovery of DON was around 35% after 4 weeks of exposure and 56% after 8 weeks of exposure and is found to be highest in the control and spiked samples. As DON was not within the original focus of the study, no measurements of any potential de-epoxydised or conjugate metabolites were carried out.

**Table 5:** Mean, standard deviation and recovery of DON in μg per absolute amount of feed, larvae and residue detected after 4 weeks of exposure and 24 h of fasting and B) 8 weeks of exposure and 24 h of fasting

	4 weeks of exposure				8 weeks of exposure			
Diet code	Feed (μg per amount feed)	Larvae	Residue (μg per amount residue)	Recovery (%) DON	Feed (μg per amount feed)	Larvae	Residue (μg per amount residue)	Recovery (%) DON
C	19.4 ± 0.7	nd	7.3 ± 0.2	37	20.0 ± 0.7	nd	14.1 ± 0.1	71
S1	19.3 ± 2.4	nd	7.1 ± 1.0	37	19.9 ± 2.5	nd	11.5 ± 0.6	58
S2	19.6 ± 0.8	nd	6.6 ± 0.4	34	20.2 ± 0.8	nd	11.7 ± 1.3	58
A1	31.9 ± 2.2	nd	10.8 ± 1.0	34	32.9 ± 2.2	nd	18.5 ± 1.3	56
A2	71.4 ± 5.6	nd	23.3 ± 1.6	33	73.5 ± 5.7	nd	39.3 ± 2.3	53
N1	97.0 ± 2.9	nd	27.9 ± 2.8	29	99.9 ± 3.0	nd	51.6 ± 1.5	52
N2	156.0 ± 14.3	nd	38.4 ± 3.3	38	160.6 ± 14.7	nd	73.9 ± 10.4	46

*nd* not detected, therefore either not present or not present in detectable levels (LOQs for DON 67.1 μg/kg in feed and 251 μg/kg in residue**)**

## Discussion

The aim of the present study was to investigate the recovery of ZEN after uptake from contaminated flour by insects of the *T. molitor* species and to monitor its impact on the larval growth. Based on the data obtained in the present study, *T. molitor* larvae have a high tolerance for ZEN containing feed. This is in accordance with several other studies which showed that *T. molitor* larvae can be grown on a mycotoxin-containing diet without increased mortality (Abado-Becognee et al. 1998; Davis and Schiefer 1982; Guo et al. 2014). The naturally contaminated diet containing up to approx. 4600 μg/kg DON and 900 μg/kg ZEN even led to an apparently higher weight gain in the larvae.

As the feed prepared from naturally contaminated grains was more protein rich compared to the other diets, larval weight gain was corrected for the elevated protein content of the feed in N1 and N2, as the availability of protein from the feed may impact on larval weight gain. Davis and Schiefer (1982) suggested the normalisation for larvae of *T. molitor* to take into account the protein effect on the growth when monitoring the effects of T-toxin exposure (Davis and Schiefer 1982). Protein contents of the diets C, A2, N2 were measured and protein content for the further diets prepared by using different ratios of the aforementioned feed were mathematically derived. ZEN spiking (S1 and S2) was assumed to have no impact on the protein content. After normalisation for the protein content, still a significant higher weight gain was found in larvae fed on a naturally contaminated diet for 8 weeks. Therefore, the presence of the fungus or other beneficial fungal secondary products seems to be responsible for an enhanced growth. Other studies showed no significant difference but a strong tendency for an enhanced weight gain when comparing *T. molitor* larvae grown on a naturally DON-contaminated diet versus a spiked or DON-uncontaminated control diet (Van Broekhoven et al. 2017). Also, the presence of aflatoxin B1 (AFB1) in feed did not show a significant difference in weight gain of black soldier fly larvae (*Hermetia illucens*) and *T. molitor* larvae when fed on either AFB1-containing feed or AFB1-free feed (Bosch et al. 2017). Guo et al. (2014) showed that *T. molitor* larvae are significantly more attracted to wheat colonised with *Fusarium* spp. *proliferatum*, *poae* and the ZEN-producing *Fusarium culmorum* compared to a control. Avoidance or preference for the infested wheat was correlated with feeding behaviour and larval weight gain (Guo et al. 2014). Eventually, such feed contains micronutrients that enables for a faster growth and better feed conversion. It might be hypothesised that during evolution, some insects like *T. molitor* have gained the capability to sense such grains infested with fungi that provide additive nutritional value. In turn, the increased feeding preference might be due to the specific capability of *Fusarium* strains to actively attract *T. molitor*. The larvae have been shown to enhance the dissemination of *F. proliferatum* by carrying the fungus in the digestive tract or adherent to the larval body (Guo et al. 2018). Our study is also in line with van Broekhoven et al. (2017) who could show that not the spiked mycotoxin alone but only naturally contaminated grain material possesses additional attractiveness for the larvae (Van Broekhoven et al. 2017).

Like previously reported for other mycotoxins such as AFB1 and DON, also the present experiment shows that in some feeding groups, more than 50% of the presumably ingested ZEN remained undetected after analysis of the insects, the unconsumed feed as well as the larval faeces (Bosch et al. 2017; Van Broekhoven et al. 2017).

For the first time, the present study included known phase I and II metabolites of ZEN and DON in the LC-MS/MS analysis. However, due to the lack of available standards for several metabolites, only qualitative analyses could be performed. Based on experience from prior work, the sensitivity for glucuronides should not differ strongly from the parent compound (Warth et al. 2012). Additionally, sulfates should be detectable with much higher sensitivity in ESI-MS analysis (Borzekowski et al. 2018). Thus, it is expected that no large quantities of ZEN phase II metabolites should have been missed during analyses. Anyway, the absence of any detectable signals of ZEN14Sulf or ZELSulf in the feeding group S1 and S2 implies that no sulfation occurs during metabolic conversion of ZEN in *T. molitor* larvae. On the other hand, the shift from the dominant ZEN14Sulf signal in naturally contaminated feed towards equal signal intensities for two distinct reductive ZELSulf metabolites compared to ZEN14Sulf in the residue samples shows that not only free ZEN but also ZEN14Sulf is converted either in the insect or due to the influence of intestinal bacteria coming into contact with unconsumed feed after excretion with the faeces.

Additionally, a higher excretion of ZEN and DON was found in the residue of the spiked samples compared to the artificially and naturally contaminated samples. Similar results were found in the study of van Broekhoven et al. (2017), who found a higher excretion of DON in *T. molitor* larval faeces for the DON-spiked diet compared to the naturally contaminated diet fed to larvae (Van Broekhoven et al. 2017).

Overall, no ZEN or ZEN metabolite accumulation was detected in any of the larval groups in this study. Furthermore, after the experimental and 24-h fasting period, no ZEN-related substances could be detected in the insects. Thus, all of the monitored substances are efficiently and rapidly excreted from the organism. Also, Guo et al. (2014) and van Broekhoven et al. (2017) did not detect any DON or DON derivatives in *T. molitor* larvae fed with infested wheat (Guo et al. 2014; Van Broekhoven et al. 2017). However, it cannot be excluded that non-detection is due to the fact that metabolites were formed which do not fall in the scope of this study.

The present study suggests that transformation of either ZEN to ZEL or ZEN14Sulf to ZELSulf but no sulfation takes place during larval metabolism. This is in accordance with a study of Camenzuli et al. (2018) who showed that α- and β-ZEL were detected in the residue of black soldier fly larvae (*H. illucens)*, while only ZEN was present in the initial feed (Camenzuli et al. 2018). Similar results were also found by Keller et al. (2018) who observed that the nematode *C. elegans* metabolised ZEN into α- and/or β-ZEL, while ZEN14Sulf was metabolised in α- and/or β-ZELSulf (Keller et al. 2018). The research performed by Kostaropoulos et al. (1996) shows that detoxification enzymes are present in *T. molitor*, which makes ZEN metabolism in larvae possible (Kostaropoulos et al. 1996). However, also other biotransformation reactions catalysed by the larvae themselves or by bacteria colonising, the larval digestion tract could be considered. Within the present study, the amount of α- and β-ZEL found in the residue is in all cases double as much for β-ZEL, compared to α-ZEL. The aforementioned is in accordance with data found in previous performed research, where it was shown that metabolism of ZEN into α- and/or β-ZEL occurs in different ratios depending on the animal species (Yang et al. 2017). It is known that α-derivatives are more abundant in dogs, pigs and turkey, while the β-derivatives are more prevailing in goats, horses, cattle and laying hens (EFSA 2017). Mammal species including humans predominantly form the conjugated ZEN or ZEL sulfates and glucuronides that appear to be absent in insects or nematodes despite the fact that, e.g. insects possess enzymes of the sulfotransferase class which would in principle enable them to for e.g. ZEN14Sulf formation (Pfeiffer et al. 2010, 2011; Zöllner et al. 2002). Further studies on the metabolism of ZEN showed that *Fusarium graminearum*, *Aspergillus niger* and *Rhizopus arrhizus* can transform ZEN into ZEN14Sulf and presented the formation of glucosides and sulfates by several *Aspergillus oryzae* strains and *Rhizopus* species (Brodehl et al. 2014; el-Sharkawy et al. 1991). For ZEN, at least it becomes obvious that the phase I metabolism shows clear similarity to other species investigated earlier while none of those animal or microorganism models had a similar outcome for the phase II metabolism shown in our feeding trial with *T. molitor*.

From the food and feed safety perspective, our data enforces the findings published for the fate of ZEN in other insect species such as larvae of black soldier fly (*H. illucens*) and lesser mealworm (*A. diaperinus*). The vast majority of ingested toxin is rapidly excreted and having in mind the common application of an at least 24-h fasting period before harvesting the larvae, the remaining ZEN as well as its metabolites should be negligible in the insects.

Although neither ZEN nor its reductive metabolites are detectable in the *T. molitor*, the intense formation of the more potent oestrogen α-ZEL might impact on the organism. It should be investigated in how far oestrogenic substances could impair the pupation of the larvae and thus negatively influence the reproduction process of *T. molitor* to avoid negative effects on the stable reproduction capability when the insect is used for feed production (McCallum et al. 2013; Roy et al. 2007).
